# Lecture on Pneumonia and Delirium Tremens

**Published:** 1840-01-18

**Authors:** W. W. Gerhard


### CLINICAL LECTURE.

### PHILADELPHIA HOSPITAL.

*Wednesday, January 8th*, 1840.

### LECTURE ON PNEUMONIA AND DELIRIUM TREMENS.

By W. W. Gerhard, M. D.

*No.* 8—*Winter Course.*

At the last lecture I brought to your notice some of the varieties of pneumonia, with their appropriate methods of treatment: to-day I will enter somewhat more fully into the subject.

The first patient to whose case I shall allude, is the man whom you saw last week, suffering with gangrene of the lung following pneumonia. He is now doing well, and is in a fair way for recovering. The other case of this lesion, which you saw some weeks since, likewise occurred as a consequence of pneumonia; but the patient had received no attention before his entrance into the hospital, at which time the inflammation had already passed into gangrene, and the physical signs proved the existence of a large cavity. No pus-secreting membrane was formed around it, but the disorganization continued to extend itself until it resulted in the patient's death. If the proper treatment had been adopted before the disease had passed into gangrene, this lesion might probably have been prevented altogether, or at least made to assume a more manageable form. The cases of pneumonia which tend to this termination are generally of an asthenic character, and therefore require particular modifications of treatment: of these I spoke more fully at my last lecture. You must not infer that gangrene of the lungs is always a consequence of pneumonia; on the contrary, it not unfrequently arises idiopathically from general causes, and does not follow inflammation. When the disease is once set in, the treatment in either case is quite similar.

In the present case, the gangrene has been circumscribed; the cavity produced by it is lined by a healthy false membrane secreting pus; and these circumstances justify us in looking for the complete cicatrization of the cavity. The proofs of the condition of things just described are found in the character of the expectoration, and the physical signs. The expectoration last week was of a dark colour, and fœtid odour, and resembled in appearance a decoction of Iceland moss; it is now mucopurulent, and without any disagreeable odour; two days since it had more or less of the characters of lymph. The cavernous rhonchus, which has been loud and distinct, is now changed into a mucous rhonchus, which is gradually disappearing. The danger, therefore, so far as regards the gangrene, is entirely passed, and we have only to fear those dangers which usually attend convalescence: the probability is in favour of a complete recovery. The treatment of asthenic pneumonia terminating in gangrene, is simple and uniform. We are to abstain from antiphlogistic measures altogether, unless the disease be complicated with pleurisy,—in which case a few cups to the thorax are allowable. Our object, on the contrary, is to support the system,—and, to this end, we should trust principally to a general plan of treatment. Quinine, in free doses, is one of the most useful remedies at our command; wine, porter, and other stimuli, may be employed, according to the circumstances of the case. These are the articles which we have mostly relied on in the present case: we have not resorted to depletion at all, for reasons already stated.

When I published some cases of gangrene of the lungs a few years since, I stated the average mortality to be about one-half. Since that time I have found the disease more fatal, the mortality having increased to nearly three-fifths. One reason of this increase is, that we generally, in hospitals, meet with the disease in its terminating stages only, as was the case in our last patient: if it were treated from its commencement, the average number of fatal cases would probably be about one-half. If an antiphlogistic treatment were practised, they would undoubtedly be much more numerous.

*Case* 2.—This is a case of the ordinary acute pneumonia, with pleuritic effusion. At his entrance into the hospital, the patient complained of pain in the right axilla, which was increased by inspiration. This pain occurring in pneumonia, is a symptom of the attendant pleurisy, and is produced by the friction of the inflamed pleura upon the opposite one during the act of respiration: inflammation of the substance of the lungs, in itself, causes little or no pain. The pain, of course, is greater or less in degree, according to the extent of the pleurisy. Sometimes the inflammation of the pleura is slight, while that of the lung itself is severe and extensive: on the contrary, we have sometimes a slight pneumonia, accompanied by a very well marked pleurisy. Of the latter sort is the case before us: this is proved by the local signs. As to the general signs, they are of very little use in enabling us to judge of the degree and extent of inflammation of the lungs; physical signs, and these alone, will inform us accurately. The local signs of pleurisy in this case, in addition to the pain, are flatness on percussion, becoming more distinct as we approach the bottom of the chest and enlargement of the right side of the thorax; these coexist with crepitus and bronchial respiration, which indicate the presence of pneumonia. These cases of pneumonia, attended with well marked pleurisy, generally terminate favourably, unless the disease should extend to both lungs; for the inflammation is of a frank, open character. The most dangerous cases are those in which the inflammation is deeply seated, particularly when it is of an asthenic grade; because we have then to apprehend its terminating in gangrene. The treatment of acute inflammatory pneumonia is very simple; it is conducted upon ordinary antiphlogistic principles.

*Case* 3.—This man was admitted into the hospital with symptoms of cerebral disorder; he had frequent attacks of epilepsy, terminating in coma. These symptoms had considerably abated, the convulsions being less and less frequent; but some days since, symptoms of pneumonia began to manifest themselves. There was no pain, because the patient's sensibility had in a great measure been destroyed by the cerebral affection; for the same reason, the pneumonia of old persons is seldom attended with much pain. Though there was no pain, there were great præcordial oppression and dyspnœa; flatness on percussion; mucous rhonchus, but no signs of the crepitant. There were also signs of bronchitis, with its ordinary cough. The countenance was yellow or icterode: this is accounted for by the fact that the inflammation had passed across the diaphragm, and involved the liver. The lower part of the lung, however, was not inflamed at the commencement: from some cause, which is not understood, asthenic pneumonia generally begins in the upper lobe; and in this case the disease first attacked the opposite lung, and has only lately extended to the right. The lips and eyelids were livid; the pulse feeble; the temperature of the skin very inconstant, being sometimes warm and pleasant, sometimes cold. These attacks of chilliness, of course, increase the danger of the patient. The termination of the case, if left to itself, would certainly be unfavourable.

We judge of the propriety of stimulation not only by the condition of the skin and pulse, but by the accumulation of liquid in the bronchial tubes, giving rise to a loose mucous rhonchus, or even gurgling, heard often at some distance from the patient. When our object is attained by bringing about expectoration, or by renovating the strength of the patient, the antiphlogistic, or the sedative practice may become proper. All that I would wish to inculcate is, that in pneumonia, even to a greater degree than in many other diseases tending naturally to a favourable termination, an exclusive plan of treatment, whether antiphlogistic or other, is an absurdity. A physician has more occasion for all his practical *tact*, or readiness of discrimination, and by a ready variation of his treatment, can retain most cases of pneumonia entirely under command.

I have already stated at the last lecture that wine is also an excellent stimulant, the best, indeed, that we can employ: wine whey is the best form for administering it. In old persons particularly, we have sometimes to use it very freely; for it is in them that this variety of disease is most frequent and most serious: in younger persons it seldom occurs, unless the inflammation has been improperly treated at its commencement. A nutritious diet is also to be allowed, to as great an extent as the patient's stomach will bear it.

There are several other cases of pneumonia at present in the wards, only one of which I shall be able to show you. All of these cases are more or less complicated with some other disease. One of these which you have just seen is complicated with gangrene; the other with pleurisy, which supervenes, indeed, in every case where the pneumonia is near the surface of the lung. A third case is of the asthenic variety, passing rapidly into suppuration. A fourth is complicated with gastritis and cerebral disorder. The patient is an old man, who was first seized with pneumonia, and afterwards with symptoms of gastric irritation; these were pain on pressure in the epigastric region, nausea, and frequent retching. The tongue was dry and red, afterwards becoming blackish; this appearance of the tongue indicated the previous existence of diarrhœa, though there was none at the time of the patient's entrance; since then, however, it has again commenced. Symptoms of cerebral disorder have likewise supervened, as subsultus tendinum, delirium, &c. Pneumonia complicated with severe inflammation of the stomach is generally fatal; cerebral symptoms are developed in almost all these cases, which, of course, increase the danger. The treatment in the case just detailed has consisted of the application of a blister to the epigastrium, and the use of small doses of opium, ipecacuanha, and calomel. A fifth case is complicated with incipient phthisis and endocarditis. In this, the dyspnœa is disproportionate to the extent of the pneumonia, and the degree of prostration. The sixth is complicated with *mania â potu*: but is convalescing.

In none of these cases has it been necessary to resort to active depletion; mild antiphlogistic and palliative means have been relied on, and in some of them, stimulation has been necessary. Unless the varieties of pneumonia be considered, and corresponding modifications in the treatment be adopted, the physician will inevitably commit many and serious errors. At the present time, as will be inferred from the preceding statement, cases of inflammatory pneumonia are unusually rare.

Of the last case which I referred to, it will be proper to give a more particular account. The patient was admitted on the 1st instant. On the 2d, I found that he presented the symptoms of delirium tremens. There were tremors over the whole body; contraction of the pupil; inability to sleep: quick pulse, and hot skin. Signs of pulmonary disease were also present; these were cough and expectoration; feeble respiration throughout the chest. There was likewise some tenderness of the epigastrium, and the tongue was coated. It appeared that the patient had been intoxicated for a week preceding his entrance, and from frequent exposure during this time, had been seized with bronchitis and pneumonia. A similar exposure in the summer would probably have produced dysentery. On the 3d, the signs of pneumonia were more distinct, and consisted of dulness on percussion, with bronchial respiration. The symptoms of mania were, however, considerably diminished. But on the 5th, the pneumonia had in a great measure subsided, while the delirium had become much more violent. Thus the two affections alternate; as the pneumonic symptoms decline, the cerebral symptoms increase, and *vice versa.* The pneumonia was treated simply with the hot infusion of eupatorium, as a diaphoretic and expectorant. This had the effect of subduing the disorder, but the delirium remained, and now required active treatment. We therefore ordered an ounce of brandy every hour. By this means the patient is nearly recovered; the pulse is soft and regular; the skin moist and pleasant; the tongue of the natural appearance, and the mind tranquil. But there yet remains a slight fulness in the right hypochondrium, which arises from the implication of the liver in the affecton of the adjacent lung.

We next have a case of simple delirium tremens. The patient has been a drunkard from his twelfth year, and he is now upwards of forty; the fit of intoxication which gave rise to his present disorder, commenced before Christmas, and continued until his entrance into the hospital a few days since. On looking at this man, the first thing that strikes your attention is a universal restlessness; the whole body is affected with tremors; when he holds out his hand, he is unable to keep it still; his tongue when protruded, is similarly agitated, but not io the same degree. Besides these tremors, last night, and several preceding nights, the patient was affected with hallucinations of mind; these are still present, but are much less manifest than they have been. As I have stated in a previous lecture, *fear* is an almost constant characteristic of these hallucinations of delirium tremens; but the fear is less of present, than of absent and imaginary objects. From this fact we derive an important lesson in the treatment of the disorder; that is, never to excite the fears of the patient, but to relieve them as far as possible by permitting him to have free intercourse with others; this will divert his mind from those terrifying objects which his imagination brings before him. The patient is always conscious of these hallucinations until his intelligence is entirely destroyed. They are most frequent and distressing when he is shut up in a cell; in company they are much less so, and more under the control of his mind.

In the consideration of this subject, the important question occurs to us, what is mania â potu, or delirium tremens? It is not inflammation of the brain or its membranes; for the symptoms of these diseases are constant; there is a permanent disorder of intellect, and a lesion of muscular power throughout many parts of the body. In delirium tremens, on the contrary, there is no such constant and decided muscular disorder; there is no rigidity or paralysis, but only agitation and inability to keep still. Nor is there any positive defect of vision, or of the other senses, other than illusions or hallucinations; they are still perfectly retained, and entirely under the control of the patient. The condition of the intellect is likewse different; in inflammation of the brain, there are rarely hallucinations, properly speaking, but a more or less complete destruction of consciousness and aberration of intellectual power; in both these respects, we observe an opposite condition in delirium tremens. This marked difference in the symptoms is explained by a reference to the pathology of the two diseases. In inflammation, there is injection of the membranes or substance of the brain, with thickening of the former, and various other organic lesions. In delirium tremens there is no organic change; the only abnormal appearance which can be detected, is an effusion of serum into the ventricles of the brain, and a preternatural moisture of the cerebral substance. This superabundance of fluid arises from the continued irritation to which the brain is subject, and the slowness with which occurs; it is not the cause of the symptoms; they are produced by the irritation, which, after it has continued for a longer or shorter period, gives rise to the effusion. The two diseases also differ in their progress. Mania â potu, like measles, scarlatina, &c., has a definite course and a natural termination; it must disappear after a certain time, unless the attack be a very severe one. No treatment is of any further use in the mild cases than to diminish the inconveniences of the disorder; any treatment which is not directed to this simple end, proves injurious by irritating and harrassing the patient.

The duration of mania â potu is not perfectly regular, but it averages about three days. This is the experience of Dr. Ware, of Boston, whose treatment in ordinary cases is almost nugatory. My own experience corresponds very nearly to Dr. W's. Very severe cases have a more prolonged course, and frequently a fatal termination. They commence, like ordinary mild cases, with tremors, followed by hallucinations and morbid vigilance; after several days they pass into the third stage, which is marked by great prostration, profuse sweating, feeble pulse, and muttering delirium. The pulse which is always a frequent but not an irritated or inflammatory one, becomes feeble, and disappears readily on pressure. There is now, as in true meningitis, a permanent destruction of intellect. These symptoms depend partly on the moisture of the substance of the brain, and the effusion into the ventricles, and partly on the nervous exhaustion produced by the previous excitement and sleeplessness.

The three stages then are generally completed in three or four days, or at most a week, and the patient either dies or gets well. It was once thought that sleep was of great importance in bringing about a favourable termination, and was, in fact, the proper crisis of the disease. This is altogether a mistake; I have seen many patients die after enjoying the soundest sleep; some, indeed, die during sleep. It is true, that when the disease declines, sleep will generally take place, in consequence of the greater quietude of the nervous system, and is so far a favourable sign; but it cannot be called the *crisis* of the disease, for it is the effect, not the cause of its declension. Hence it is often injurious to force sleep by means of opiates.

In the treatment of delirium tremens, the indication is to reduce the irritability of the nervous system. As the disease has, like measles, or small pox, a natural termination, there are various plans of treatment, under which the patient will get well; indeed, in most cases, recovery will take place, though no remedy whatever be employed; nature restores the order of the functions. The disease is very commonly treated in this way at Boston, and from time to time I have pursued in mild cases either a simply expectant treatment, or have directed very simple remedies. My colleague, Dr. Dunglison has treated many cases in the same manner.

Emetics have frequently been employed for the cure of this disorder. They act by producing relaxation and diaphoresis and in some cases this practice succeeds very well. But in other cases, (especially in that sort which is not unfreqently met with in private practice, where the disorder is brought on, not by a fit of intoxication, but by a long course of free drinking,) emetics may do a great deal of harm; instead of tranquillizing the system, they sometimes produce a great deal of prostration, which in some cases that I have seen, has undoubtedly been the cause of death. Tartar emetic is particularly liable to this objection.

Of the various other remedies employed in the treatment of delirium tremens, opiates have probably received most attention. I formerly used these remedies in almost every case, though not in as large doses as some of my brethren; but when I was a resident physician in this hospital, we were directed to give opium in very large doses,—frequently as much as four grains every two or three hours, until sleep was procured. The patients, for the most part, got well under this treatment; but in estimating the value of a particular plan of treatment, we ought to consider the proportional success of this and other plans. A comparison of this sort will prove that opium is not the most effective remedy in mania â potu. In conjunction with this remedy, certain hygienic regulations were also enforced at the time to which I have alluded. The patients were locked up in cells, and if very disorderly, that is in every severe case, they were confined in a straight jacket, or tied in bed, with gloves and straps.

The practice of the hospital has never been to give opium to the exclusion of other remedies; it was always the custom to use cups and cold applications to the head, purgatives and various other remedies, when they seemed necessary. From time to time a change would be made in the practice, and the affection would either be treated upon impirical grounds, in accordance with the varying symptoms, or the emetic practice would be pursued.

But the plan of treatment, by opiates and confinement, is the one that was almost universally practised in Philadelphia several years ago, with variable results. In my own practice I have gradually diminished the quantity of opium which I formerly gave, and for some time past have not used it at all. Instead of it, I have relied in bad cases upon the stimulant treatment which was always followed in some parts of New England, especially by Dr. Tully of New Haven; that is, the use of stimulating remedies, particularly alcoholic liquors. These articles I first employed in conjunction with opium, or prescribed them without opiates, in two different conditions; 1st, in the slighter cases, or those of incipient delirium tremens; or 2dly, in the severe cases where opium had been employed but was followed by distress of mind and stupor. But at present I use them singly. This treatment has diminished the mortality of the disease. The change which I have adopted in the hygienic rules, has also contributed very decidedly to this result. Instead of confining the patients, I let them walk about and enjoy the company of others as much as they choose: merely taking care that some one should be near them to prevent accidents. I was led to this change by observing that the hallucinations which attend the disorder were more distressing when the patients were in a state of confinement than when they were allowed to walk about as much as they wished. As I have already remarked, they are capable of controlling these hallucinations, until the intellect is entirely destroyed; and they can do so the more easily when they are surrounded by objects which may serve to engage their attention. Confinement always irritates them, and increases their ravings, so that the third stage, in which the intellect is entirely destroyed, is apt to be brought on very speedily. I have very often tested this by a simple experiment; a man who was confined to his bed by a straight jacket or something of the kind, I have frequently directed to be dressed, have soothed him by conversation, and after requiring a promise that he would conduct himself with propriety, I have very seldom found reason to be dissatisfied with the result. On the contrary, the disease would almost invariably become milder, and the necessity of confinement cease. It is true that confinement is often necessary at night, from the impossibility of always providing a sufficient number of attendants. I therefore (with the exception just stated) allow the patient to have full liberty, the only restraint being the presence of the keeper: sometimes, also, I direct them to be set at work, which serves still farther to distract their attention.

The proportional mortality under the two plans of treatment which I have detailed, is represented in the following summary, comprising the number of cases treated amongst the men for the space of $5\frac{1}{2}$ years—that is, from the 20th of May, 1834, to the 13th of November, 1839. The whole number of cases admitted for delirium tremens, or intemperance, which was expected to terminate in delirium tremens, was 1241. Of these, there were 1198 whites, and only 43 men of colour. Of the whole number, 708 were decided cases of delirium tremens, 60 were slight cases, and 430 cases of mere intemperance. Of the latter, some terminated in decided delirium tremens, and others proved fatal from diseases (such as pneumonia) contracted during the fit of drunkenness, for which they had been sent to the lunatic asylum. So that this class furnishes a considerable number of bad cases. Of the whole number, 121 cases proved fatal. That is, a fraction less than one in ten.

In the first year, from May 1834, to the date 1835, the number of admissions was 141; of these, 18 died; that is, rather more than one in eight. In the second year the number of cases was 211, the deaths 24, or a little more than one in nine. The third year, in 301 cases there were 47 deaths, a much larger proportion than in preceding years, one in 6 19-47ths, but depending upon an accidental cause; that is, the occurrence of an epidemic of typhus, which attacked many of the debauched subjects of intemperance: some of them were sent to the lunatic asylum as labouring merely under the effects of intemperance, and could not be afterwards removed to the proper ward.

In the fourth year, beginning May, 1837, of 206 cases, 19 only proved fatal, that is about one in eleven. This was a decided amelioration, and coincides precisely with the epoch at which the change of practice was introduced.

In the fifth year the mortality went on diminishing, and was less than one in twenty-six; or of 274 cases, 9 only were fatal; and amongst these cases, the mortality was certainly greatest in those which were treated chiefly according to the method formerly pursued at the hospital.

Finally, in the six months, ending November, 1839, the mortality was only one in $33\frac{3}{4}$, that is, 4 cases out of 135; and of these four, one entered moribund, and was not, therefore, treated in the hospital; another had inflicted upon himself several fractures and other injuries, by leaping from a third story window, in a fit of delirium tremens, previously to his entrance. The others, it is believed, were also complicated cases.

From the preceding statement it appears that the mortality has progressively diminished under the modified plan of treatment which has been introduced. And as some of the fatal cases recorded in the table proved so in consequence of accidents, and of other diseases developed while the patients were under treatment for delirium tremens, the mortality may be stated as at the minimum. If the enumeration included only those who really died of this disease, when treated in the hospital, it would probably be found that the mortality does not exceed one or two per cent., which must always be expected from various accidental circumstances not connected with the disease. As I have already stated, we employ, in our treatment of this disease, nervous and arterial stimulants, (to use the classification of Dr. Wood.) We often employ assafœtida, camphor, capsicum, and carbonate of ammonia, generally in some such prescription as the following:—Carb. ammon. ʒj, liq. anod. Hoffman ℥j, in ℥v of lac. assafœtida, or either of these alone, so that the patient takes ʒss or ʒj of Hoffman's anodyne, and five or six grains of carbonate of ammonia, every hour or two. The tinctures of capsicum, and valerian, or capsicum in substance, are also administered with advantage. If these fail, we resort to alcoholic stimuli, particularly brandy; we give it in various quantities,—sometimes an ounce every hour, sometimes the same quantity once or twice a day only. In mild cases it is not necessary to resort to brandy; and it is better to abstain from its use, both on grounds of morality and of utility, unless the disease be severe, and then it must be given in efficient doses. At first sight, it may seem absurd to give alcohol for the cure of a disease produced by alcohol; but the propriety of the plan becomes evident when we recollect the mode in which delirium tremens begins. After a night of drunkenness, the man wakes in the morning in a state of misery from distressing "horrors." He understands perfectly what is to be done for their removal; he resorts to the bottle, and experiences the desired relief. This routine of intoxication at night, and horrors in the morning, may be continued for a week or a fortnight. At the end of this time, the drunkard either "tapers off," as it is termed, by drinking a less and less quantity every day, or he is obliged to go to the hospital. Sometimes he takes the latter step, from want of money wherewith to purchase the remedy upon which he has been in the habit of relying for the cure of horrors; in this extremity he has to seek medical advice for their relief. At other times he is prevented from drinking to the usual excess, by the supervention of some disorder brought on by his excesses; this may be either irritability of the stomach, or irritation of the brain, headach, &c., showing a tendency to meningitis. In either case, the man is prevented from resorting to alcohol for the relief of his horrors, and delirium tremens is developed; he therefore comes to the hospital. Now, in the treatment of delirium tremens, we only imitate the practice of the drunkard, which his experience tells him to be so successful. But we do not use alcoholic stimuli so freely as he would; we give them in small doses, so that no bad effect is produced by them, while the disease is allowed to pass off gradually. When this end has been attained, the patient generally falls asleep, because the disorder which prevented him from sleeping has ceased. I have already explained to you, that sleep, of itself, exercises but little curative influence.

Opium need not be abandoned: there are many circumstances, and certain stages of the disease, where it comes in admirably, and is free from risk. This is especially the case when the patient appears drowsy; the disease has abated, but not sufficiently to allow the patient to sleep. A dose of one or two grains of opium, repeated once or twice at night, then, may bring about the desired effect by removing the nervous excitement still remaining, and allowing the natural termination of the disease to take place. All that I would wish to insist upon is, that the practice of forcing sleep by large and especially by increasing doses of opium, is not, I believe, free from danger, and certainly is less successful than other modes of practice.

It is true, many of the dangers attending the use of opiates may be obviated by extraordinary vigilance, watching carefully each dose; and you will find this caution much insisted upon in an excellent memoir published some years since by a physician of this city, who had had much experience in the management of this disease. But this caution is not always practicable, and we therefore are on the safe side in restricting the doses of the remedy, and its frequent repetition. At our hospital, the resident physicians, to whose care the cases of delirium are more immediately confided, have been equally intelligent, the superintendent is the same, there is no change of circumstances, hence we must look for the cause of the difference in the results to the modifications of the treatment. Upon analysis, we have found them to be a more careful removal of exciting causes, by allowing the patient as much liberty as is consistent with safety, and in cases which are not tending rapidly to a natural cure, replacing the excessive excitement of alcohol by the same remedy, given in graduated doses, or by other stimulants, which act less directly on the brain, and are therefore less active, probably less useful agents. You perceive that opium is by no means objectionable in certain cases,—neither are cups or cold applications to the head, nor purgatives, when the disease is attended with flushed face and an injected eye, and other symptoms of active engorgement to the head. In these latter cases, the congestion of the brain and the vascular excitement are more important than the milder derangement of the nervous system constituting the proper delirium tremens. In a case of this kind I once cured a patient by a single bleeding of twenty ounces, and you will often meet with such instances; while the very same cases may require stimulants soon after the depletion.

The inference, then, is, that in delirium tremens, most cases will get well under the influence of the same law as that regulating the termination of other limited diseases. This natural termination takes place most certainly and quickly when you do not subject the patient to solitude during the day, and to complete darkness at night, which increase the hallucinations under which he labours. Cheerful society, distraction of mind, and, when practicable, labour, are most useful adjuncts in the treatment of the disorder, or may conduct the disease more comfortably to its natural termination. Above all, these precautions prevent the development of many severe cases which then diminish singularly in number.

Though various remedies are at times used in this hospital, which have their advantages in particular cases of delirium tremens, none can be regarded as strictly curative, unless it exercises a stimulant influence, which may, to some degree, replace the action of alcohol, or substitute for the irregular use of this remedy its prescription in small and rapidly diminishing doses. The great majority of cases do not require this mode of treatment,—they will, indeed, recover without any treatment; hence it is a duty to resort to a mode of treatment to which some objections exist only under circumstances in which the disease would become dangerous, or be needlessly prolonged. There are other cases in which the proper delirium tremens is slight, but the irritation of the stomach and brain are of more severity, which would not be benefited, but, on the contrary, be much injured by this treatment.

I have not observed that patients treated by alcohol, relapse into their former habits more rapidly than others. If the cure is not complete, if there remains any restlessness, the patient will almost surely relapse, treat the case as you may. If it be complete, a return to habits of intoxication depends upon causes which arise from various circumstances not under the control of the physician, who is called to prevent the mischief resulting from habits of intemperance. The eradication of the disease will doubtless follow the improvement in the habits of the people which is gradually advancing; and the continued efforts of many minds will bring it about in the only practicable way, by moral persuasion, and rational conviction of the inevitable consequences of excessive indulgence in ardent spirits.

From the great length of this lecture, I am reluctantly obliged to omit the discussion of the various circumstances influencing the development of the disease in men of the same habits, as well as the more exact determination of the cases in which alcohol is necessary. Of the accuracy of the statements you may be well assured, for they are compiled directly from the official registers. My own convictions have been gradually formed; and in restricting so much the use of a remedy formerly almost exclusively relied upon, I am influenced solely by the belief that other modes of practice are more successful.

